# Guiding the development of sustainable nano-enabled products for the conservation of works of art: proposal for a framework implementing the Safe by Design concept

**DOI:** 10.1007/s11356-019-05819-2

**Published:** 2019-07-06

**Authors:** Elena Semenzin, Elisa Giubilato, Elena Badetti, Marco Picone, Annamaria Volpi Ghirardini, Danail Hristozov, Andrea Brunelli, Antonio Marcomini

**Affiliations:** Department of Environmental Sciences, Informatics and Statistics, Ca’ Foscari University of Venice, Via Torino 155, 30172 Venice, Italy

**Keywords:** Sustainability, Safe by Design, Conservation science, Nanomaterials, Chemical safety, Safe innovation, Decision support

## Abstract

Nanotechnology provides innovative and promising solutions for the conservation of cultural heritage, but the development and application of new nano-enabled products pose concerns regarding their human health and environmental risks. To address these issues, we propose a sustainability framework implementing the Safe by Design concept to support product developers in the early steps of product development, with the aim to provide safer nano-formulations for conservation, while retaining their functionality. In addition, this framework can support the assessment of sustainability of new products and their comparison to their conventional chemical counterparts if any. The goal is to promote the selection and use of safer and more sustainable nano-based products in different conservation contexts. The application of the proposed framework is illustrated through a hypothetical case which provides a realistic example of the methodological steps to be followed, tailored and iterated along the decision-making process.

## Introduction

Nanotechnology provides innovative and promising solutions to contrast degradation processes of artistic materials and achieve long-term conservation of cultural heritage (Baglioni and Chelazzi 2013). This is particularly beneficial in the case of the complex and often unstable materials used by contemporary artists. Some issues of conventional techniques for remedial conservation and restoration can be overcome by nano-based formulations specifically developed for the controlled cleaning of surfaces, such as nanofluids composed of micelles or microemulsions (Chelazzi et al. 2018) applied, for example, to frescos (Baglioni et al. 2014) or to graffiti (Giorgi et al. 2017). Nanoscience also provided valuable solutions for polymer film dewetting by water/surfactant/good-solvent mixtures (Baglioni et al. 2018) and for the consolidation and stabilisation of different artistic surfaces like cellulose (calcium hydroxide nanoparticles in Poggi et al. 2014), painting canvases (combined nanocellulose/nanosilica in Kolman et al. 2018) or bronze (layered double hydroxide nanoparticles filled with corrosion inhibitors in Salzano De Luna et al. 2016), while innovative nano-based solutions are still under development for plastic surfaces (Shashoua 2016).

However, the development and application of new nanomaterials and technologies for the conservation of cultural heritage (where, with the term conservation, we refer to remedial conservation and restoration as defined by the International Council of Museums—committee for conservation (ICCOM-CC 2008), thus excluding preventive conservation) pose several concerns regarding their human health and environmental risks (Eason et al. 2011). Therefore, there has been growing research into the health and environmental implications of these nano-enabled formulations. Some examples of recent European research projects focusing on these aspects are NANOforART, HEROMAT, NANOMATCH, NanoCathedral, NANORESTART and InnovaConcrete.

Even without specifically considering the inclusion of nanomaterials in conservation products, the use of chemical substances and mixtures, the fact that restorers are often operating in indoor environments and the high variability of both magnitude and duration of exposure (due to a high variability in the performed activities) can result into serious chemical health risks, ranging from mildly irritative changes in the upper airways induced by nuisance dust to the carcinogenic effects of certain paints and pigments (Varnai et al. 2011; D’Angelo and Accardo 2012). Moreover, the disposal of chemical waste and the limited application of emissions control could represent a threat to the natural environment (D’Angelo and Accardo 2012). However, despite an increasing attention in recent years to “green restoration” of cultural heritage through developing safer and “greener” technologies (Balliana et al. 2016), very few works performed actual assessment of health and environmental risks by means of experimental or modelling techniques (Tedesco et al. 2015; Ferrari et al. 2015; Turk et al. 2017; Pineda et al. 2017; Franzoni et al. 2018; Mauko Pranjić et al. 2018), mainly focusing on life cycle assessment (LCA) of consolidants for the conservation of immovable cultural heritage (e.g. historical buildings), and an overarching framework for assessing the sustainability of the nano-enabled products used in the conservation of cultural heritage is currently lacking.

Such a framework should explicitly incorporate the Safe by Design (SbD) concept, which offers a sound strategy for ensuring the safety of new products in the early design stage, while retaining their performance and functionality in commercially viable ranges (Gottardo et al. 2016; Noorlander et al. 2016; Kraegeloh et al. 2018). The implementation of this concept in the domain of conservation of cultural heritage is essential as this field has been traditionally driven by technical requirements (e.g. compatibility with artistic materials, controllability and selectivity of the treatment) (Baglioni and Chelazzi 2013), while the safety and sustainability aspects have been neglected. Moreover, the application of a SbD concept can limit the need to find better alternatives in the future, once the products are ready to enter or are already in the market (e.g. Giubilato et al. 2016; Aschberger et al. 2017). The SbD concept will be further presented and discussed in “Background: the Safe by Design concept”.

In the scientific literature, the safety of products is pinpointed as a key element in the overall sustainability of nanotechnology (Dhingra et al. 2010; Mulvihill et al. 2011; Schulte et al. 2013; Hjorth et al. 2017), where the concept of “sustainable nanotechnologies”, although increasingly used to guide decisions on technological development, has not been clearly defined (Subramanian et al. 2014). There is a general consensus on considering material price, carbon footprint, resource scarcity, ecotoxicity or human health effects among sustainability concerns (Babbit and Moore 2018; Linkov et al. 2009), but a clear operationalization of a strategy for sustainable nanotechnology innovation has just started to emerge as a result of a nascent dialogue among stakeholders from industry, academia and regulation (Falinski et al. 2018; Babbit and Moore 2018; Cinelli et al. 2016; Subramanian et al. 2016; van Harmelen et al. 2016; Subramanian et al. 2015; Linkov et al. 2015; Falkner and Jaspers 2012).

From a regulatory point of view, in Europe, the REACH (European Commission 2006) and the CLP regulations (European Commission 2008) represent the references for the safety assessment and management of nanomaterials for conservation when they occur as substances or in mixtures. These pieces of legislation provide the boundaries for the chemical safety assessment of new formulations along their life cycle and set the ground for a more comprehensive approach to cope with the several issues related to the sustainability of the new products, where information on environmental, economic and social dimensions are integrated according to the so-called triple bottom line (TBL) approach (Elkington 1999).

To address this gap, we propose a framework to inform the design of sustainable nano-based products for conservation of art, taking into account the current EU legislative context as well as the specific features of the innovation process in the cultural heritage conservation field, which demands a high interaction between the product developers and the restorers (Ormsby et al. 2016; Baglioni et al. 2015). This framework was developed in the frame of the NANORESTART (NANOmaterials for the REStoration of works of ART) EU project with the aim of assisting formulators in the early steps of development and refinement of these new products. In addition, to support efficient innovation pathways, the framework is expected to facilitate communication with conservators for selection and use of safer and more sustainable nano-based products in different conservation contexts.

## Background: the Safe by Design concept

The development of a sound approach to tackle sustainability of nanomaterials, including Environmental Health and Safety (EHS), for artworks conservation cannot overlook the SbD concept, which gained an increasing attention in recent years in European FP7 and H2020 research projects focused on engineered nanomaterials (Hjorth et al. 2017). The basic idea to implement the “safety by design” consists of anticipating potential human or environmental impacts of a new material or product, with the objective of modifying its design to avoid undesired properties while keeping the required functionalities. Since it was developed in a regulatory context, regulatory requirements, such as those included in REACH, Biocidal Products Regulation or Occupational Safety information requirements, are at the core of SbD (Kraegeloh et al. 2018).

The most comprehensive definition of the SbD concept for nanomaterials has been so far developed within the NANoREG and NanoReg2 EU projects, where SbD has been presented as an approach to transfer the precautionary principle into practical use, by considering the functionality of a nanomaterial and its toxicity/safety in an integrated way (Gottardo et al. 2016; Noorlander et al. 2016; Kraegeloh et al. 2018).

The Cooper Stage-Gate innovation model (Cooper 1990, Cooper and Robert 2011) was chosen as the backbone for the SbD concept, with the aim of incorporating SbD in already-used structured innovation management processes. Stage-Gate is an industrially standard systematic approach that divides the innovation process into a predefined set of stages (usually five), moving from new product ideas to launch to market and beyond. Each stage includes specific activities (e.g. preliminary market assessment, detailed financial analysis and laboratory work), and the advancement from one stage to the following one is regulated by a gate, where the innovation project is judged according to a set of criteria. The output of each gate is a Go/Kill/Hold/Recycle decision about the project and an action plan for the next stage.

The gate decisions, in the SbD concept, depend also on safety considerations and risk potentials associated with the development, manufacturing, use and disposal of the new nanomaterials. For this reason, currently used management processes for innovation risks, EHS, regulatory requirements and safety data handling have been integrated in the SbD concept (Noorlander et al. 2016). The innovation risk management process deals with all risks considered “consequences of uncertainties” according to ISO 31000 standard (ISO 2009) and includes risk assessment and risk treatment phases. EHS management process focuses on the screening and management of occupational health hazards and possible environmental impacts related to the innovation project, adopting a product life cycle perspective which can foresee also the application of typical LCA tools. Regulatory management requires the identification of applicable regulations and their implementation using appropriate data, including the relationship with regulatory authorities. Finally, safety data management process specifically refers to the collection and/or generation of data necessary to implement the aforementioned processes.

The SbD concept was also combined with the Regulatory Preparedness (RP) concept, which is based on promoting early interactions between innovators and regulatory authorities with the goal of sharing expertise for an early identification of uncertainties and potential risks and for acquiring the necessary knowledge to meet all regulatory requirements in due time. Both concepts (SbD and RP) were finally embedded in the Safe Innovation Approach (SIA) which is expected to be implemented through an intense interaction and collaboration between industry and regulators since the very beginning steps of the innovation process (Kraegeloh et al. 2018).

Even if weaknesses and criticalities in the version of the NANoREG/NanoReg2 SbD concept have been identified (Hjorth et al. 2017), nonetheless this concept and its incorporation into the SIA currently represent the most comprehensive approach to the safer development of new nanomaterials and should therefore constitute a fundamental reference also in the development of new nano-based products for conservation.

## Methods

**Fig. 1:**
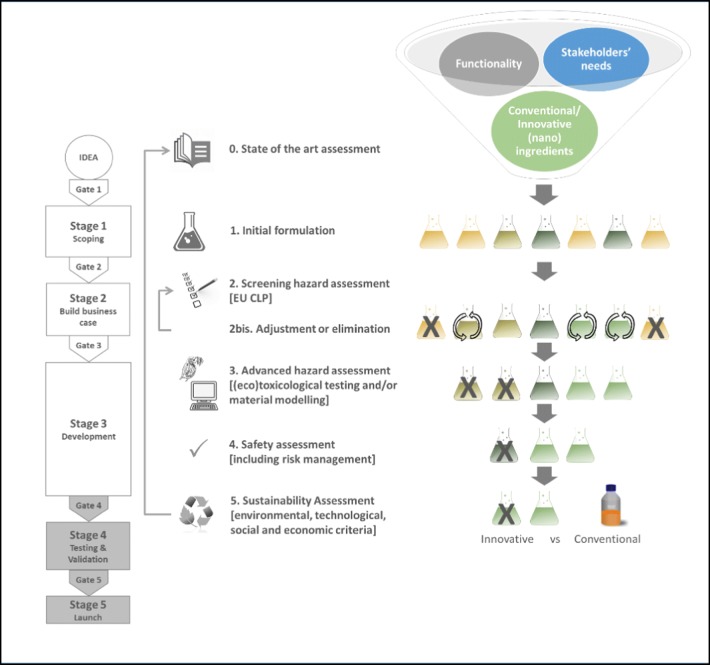
Sustainability assessment framework implementing the SbD concept mapped against the Stage-Gate innovation process

The proposed stepwise sustainability assessment framework is depicted in Fig. 1, against the Stage-Gate innovation process briefly described in “Background: the Safe by Design concept”. It covers the first part of the value chain, from “basic research” to “materials R&D and processing” up to “applied R&D” (as described by Noorlander et al. 2016), thus not including industrial manufacturing, transport, use and end of life, although use scenarios are roughly identified. It allows the comparative assessment of new nano-based products against conventional products (if they exist) and focuses on application and post-application stages, since the manufacturing stage cannot be considered until the industrial upscale of the new products has been completed. Each step of the sustainability assessment framework is described in the following paragraphs**.**

### Step 0: State-of-the-art assessment

Before starting any innovation process, a detailed assessment of the state-of-the-art in a specific field must be performed in order to discover opportunities and to generate new product ideas (the so-called stage 0 of the Stage-Gate innovation process). Specifically, product developers should at least know (i) which are the stakeholders’ needs in terms of functionalities (e.g. cleaning of paints, coating of metal substrates for indoor/outdoor conditions), (ii) if there are products already on the market covering a specific functionality, as well as if there is any environmental, economic or social issue associated with them and (iii) whether there is any promising ingredient in the nanoform they would like to use. For this latter, the application of the adapted Ashby’s framework for sustainable engineered nanomaterials (ENMs; Falinski et al. 2018; Babbit and Moore 2018) could be considered to support the selection of appropriate ENMs to be incorporated into the innovative formulations.

In this step, technical (e.g. compatibility with artistic materials), environmental (e.g. toxicity of ingredients), social (e.g. ethical criteria such as reversibility of treatment) and economic (e.g. cost of the final product) criteria are broadly considered by product developers in order to generate a first idea of the innovative product.

### Step 1: Initial formulation

According to the results of the previous step, product developers propose a set of innovative formulations for a specific functionality, taking into account the final goal of developing safe (green) products (stage 1). This means that, in this step, mainly technical and environmental criteria are considered, although some social and economic aspects could be already in the background.

### Step 2: Screening hazard assessment (EU CLP)

In this step, the environmental performance of innovative formulations proposed in step 1 is checked by a screening hazard assessment. For this purpose, the EU CLP self-classification approach for mixtures is applied (European Chemicals Agency 2017) in order to derive the health (H) and environmental (ENV) hazards potentially associated with the innovative products in a quick and inexpensive manner (stage 2). At this level, usually “test data on the mixture itself are not available for a mixture, therefore bridging principles and weight of evidence determination using expert judgement for all the necessary H and ENV hazard assessments may not be applied. In these cases, classification must be based on calculation or on concentration thresholds referring to the classified substances present in the mixture” (European Chemical Agency (ECHA) 2017). According to ECHA (2017), concentration thresholds are “generic cut-off values i.e. the minimum concentrations for a substance to be taken into account for classification purposes, and generic concentration limits (GCL) i.e. the minimum concentrations for a substance which trigger the classification of a mixture if exceeded by the individual concentration or the sum of concentrations of relevant substances (where the individual substance concentrations can be ‘added’ to each other in a straight forward way)”. As an example, “the generic cut-off value for a skin irritant substance which is present in a mixture is 1 %. A GCL of the skin irritant substance above or equal to the concentration limit of 10% triggers classification of the mixture for skin irritation. However, at ≥ 1 % and below 10 %, the substance may still contribute to the classification of the mixture as skin irritant. This because the concentration would be taken into account if other skin corrosive/irritant substances are present in the mixture below the relevant generic concentration limits” (ECHA 2017).

To apply such self-classification approach, the following data should be collected: (i) the list of ingredients included in each formulation, (ii) their safety data sheets (SDS) including the classification for H and ENV hazards according to the EU CLP regulation and (iii) the percentage (*w*/*w* as single value or range of values) of each ingredient in the formulation. In some cases, the SDS of a specific ingredient could not be available because, for example, it was newly synthetized by the product developer or it is in a specific nanoform while the SDS is available for the bulk counterpart only. In these cases, one can decide to indicate that H and ENV hazards are unknown for a specific percentage of the formulation (i.e. the %*w*/*w* of the specific ingredient) or to use the SDS of the ingredient bulk form. The results of the screening hazard assessment are communicated to the product developers along with an explanation of the thresholds applied for each hazard and the indication of how specific hazards could be avoided (e.g. reducing the %*w*/*w* of a specific ingredient or substituting it with a safer alternative).

### Step 2bis: Adjustment or elimination

According to the results of the previous step, product developers can adjust the initial formulation to reduce its hazard or decide to discard it in case an adjustment to the composition would negatively impact its technical performance and functionality. Step 2 can be iterated several times (i.e. subsequent adjustment and self-classification of the formulations) according to the needs of the product developers. The result is a reduced number of formulations for which a good environmental performance (where “environmental” refers to the “environmental pillar” of sustainability although hazards for both human health and the environment are checked) is demonstrated through a theoretical approach (i.e. self-classification for H and ENV hazards according to CLP regulation) (stage 2).

### Step 3: Advanced hazard assessment: integrated testing strategy

The environmental performance of the selected formulations is further checked in this step through an advanced hazard assessment in which computational (e.g. in silico models) as well as experimental (e.g. in vitro and in vivo (eco) toxicological tests) approaches could be adopted, according to an integrated testing strategy (stage 3).

An integrated (or intelligent) testing strategy (ITS) is a hierarchical, resource-effective testing scheme consisting of a set of decision nodes, allowing for taking different routes for information gathering and inference for decision-making about a chemical’s hazard or risk (Hengstler et al. 2006; Van Leeuwen et al. 2007). ITSs emerged in the mid-nineties from several research initiatives examining how to combine different testing and non-testing methods (including in silico, in vitro, in vivo and omics methods) in order to reduce, refine and replace animal testing of chemicals (3Rs principle) (Blaauboer et al. 1999; Hakkinen and Green 2002; Salem and Katz 2003; Vermeire et al. 2007) and had a considerable expansion after the introduction of the REACH regulation in 2006 (Jaworska et al. 2010). As reported by the NanoSafety Cluster Working Group (WG) 10 (Oomen et al. 2014), integrated approaches to testing and assessment (IATA), in the literature also referred to as ITS, are required for an adequate assessment of the impact of nanomaterials (NM) on human health and environment. They should (1) stand in line with current EU guidance on NM safety testing, (2) consider real-life exposure situations in order to assess toxicity of relevant form(s) along the NM life cycle and (3) include possibilities for the grouping of NM (i.e. by waiving tests based on categorisation of NM or by providing test results relevant for grouping). A comprehensive IATA is currently being developed by the NanoSafety Cluster WG 10 and should be ready by 2020 (Oomen et al. 2014). Meanwhile, Oomen et al. (2014) presented a first proposal of integrated approaches for NM toxicity and ecotoxicity testing and assessment in which a tiered structured is adopted to move from basic or general testing (e.g. in vitro tests for short-term toxicity; standardised short-/long-term test systems with laboratory organisms for ecotoxicity and additional endpoints such as enzymatic effects and functional genomics to predict NM ecotoxicity) to specific testing (e.g. in vivo tests for establishing general concepts for NM toxicity, environmental simulation studies for ecotoxicity). In this process, relevant support is provided by (1) the possibility of grouping NM (and therefore to waive some tests) and (2) the identification of main exposure paths (thus avoiding unnecessary testing).

Since these considerations are valid also for ITS not targeted to NM (Jaworska et al. 2010), they can be extended to the case of assessing mixtures containing NM, where the type and percentage of a specific NM in the final composition could affect its behaviour (e.g. whether particles are released along the life cycle) and overall toxicity. In this context, the applicability of computational approaches (e.g. read-across) as well as the clear identification of relevant exposure paths should be verified on a case-by-case basis.

Regarding the latter, information about relevant exposure scenarios for both human health (i.e. occupational and public health) and the environment (i.e. technical compartments such as waste systems and environmental compartments such as soil and water systems) along the life cycle of the innovative products should be collected or generated. Specifically, in addition to exposure measurements in occupational settings (Gherardi et al. 2007), data/information on product degradation and/or release throughout their life cycle should be collected, to evaluate medium- and long-term behaviour of nano-based products (Zuin et al. 2014).

### Step 4: Safety assessment

The results of exposure and hazard assessments performed in steps 2 and 3 are here combined to derive conclusions on the safety of the formulations in each life cycle stage and identify any hotspot (Gottardo et al. 2017). Risks to human health (for workers) or environmental compartments can be estimated through qualitative or semi-quantitative (e.g. control banding tools for occupational exposure scenarios), or quantitative methodologies, depending on the typologies of hazard and exposure information and data available from previous steps (Hristozov et al. 2016). Sources of uncertainties in the risk assessment process must be identified and, when possible, uncertainties should be quantified (Hristozov et al. 2018; Pang et al. 2017). In addition, in this step, suitable risk management measures (RMMs) for the relevant scenarios are selected from those showing efficacy in controlling nanomaterials (e.g. gloves, filtering masks and suitable ventilation as reported in Oksel et al. 2016) and are communicated to product developers and restorers for a safe manufacturing and application of the selected products (stage 3).

### Step 5: Sustainability assessment

In addition to the results from the advanced hazard and safety assessments (steps 3 and 4), to reach stage 4 of the Cooper innovation process, one should provide validation also of the customer acceptance, the economics and the social implications of the product. Therefore, in this step, sustainability is assessed by integrating information related to environmental, economic, social and technical aspects, through multi-criteria decision analysis (MCDA) methods (Giove et al. 2009). Results allow to rank innovative formulations as well as to compare them to relevant conventional products, if any, and therefore decide whether to proceed later with a pilot industrial upscale. Since the framework is aimed at guiding the design of formulations early in their development at laboratory scale, advanced tools such as LCA (ISO 2006a, 2006b) are not included in the sustainability assessment step under the environmental pillar or under the economic and social pillars as life cycle costing (Swarr et al. 2011) or social LCA (Petti et al. 2016), respectively. However, as more detailed information become available in moving from laboratory scale to industrial production, the framework can be iterated, and tools like the life cycle–based two-tiered SUNDS (Subramanian et al. 2016) can be adopted to support both steps 4 and 5.

## Hypothetical case study

In this paragraph, an example of application of the sustainability assessment framework implementing the SbD concept is provided by considering the design of a hypothetical innovative nano-based consolidation system for contemporary works of art (step 0). The new system should be able to tackle the peculiar instability and variability of complex materials used by contemporary artists and must be environmentally friendly and sustainable in all its life cycle stages (with special attention to application and post-application stages). Moreover, the product developer is considering feasibility, long-term impact on conservation, material costs and potential for industrial scalability.

**Fig. 2:**
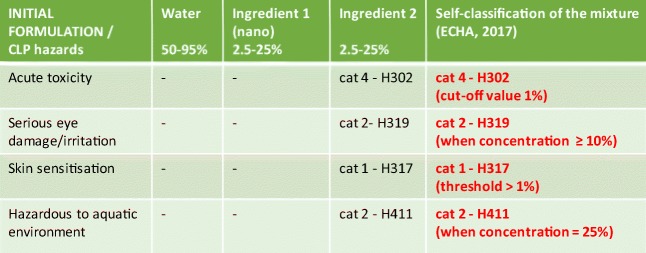
Results of steps 1 and 2: screening hazard assessment of the initial formulation (IF)

The composition of the initial formulation (IF) is provided as concentration of each ingredient in terms of ranges of values (%*w*/*w*), as depicted in Fig. 2 (step 1). In addition, SDS are provided for the two ingredients (only ingredient 1 being in nano-form), thus allowing the self-classification of the mixture according to CLP guidance (European Chemical Agency 2017). Obtained results (reported in the rightmost column in Fig. 2) are communicated to the product developer along with the thresholds leading to that classification (step 2), with the aim to provide a guidance for subsequent adjustment of formulation’s composition. Specifically, in this case, the suggestion is to reduce the concentration of ingredient 2 (the one leading to the classification of the IF for acute oral toxicity, eye irritation, skin sensitisation and chronic aquatic hazard) below 25%, thus avoiding the aquatic toxicity, or 10%, additionally avoiding eye irritation, or even 1% (which will result in no hazards).

**Fig. 3:**
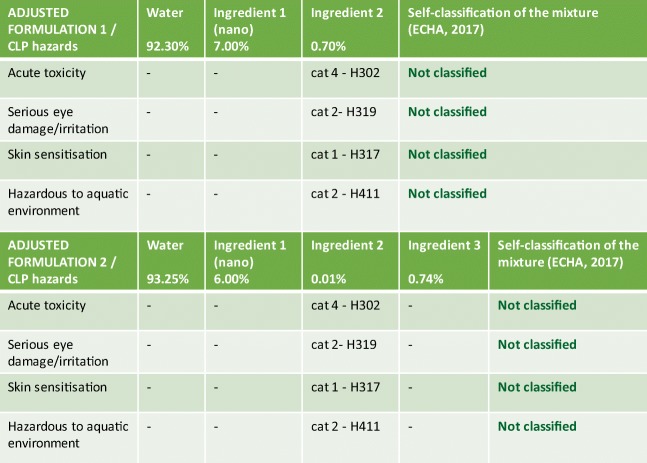
Results of step 2bis: screening hazard assessment of adjusted formulations (AF1 and AF2)

According to the provided information, the product developer further works on IF and succeeds in the development of two adjusted formulations: AF1 and AF2, by including only 0.70% (*w*/*w*) and 0.01% (*w*/*w*) of ingredient 2, respectively, and by adding the new, not hazardous, ingredient 3 in AF2. The self-classification of both formulations is reported in Fig. 3 (step 2bis).

At this point, AF1 and AF2 represent the most promising innovative nano-based consolidants to be further checked according to a tiered integrated testing strategy (ITS) in the advanced hazard assessment (step 3). Although both AFs contain a low percentage of nanomaterials (NM), exposure to nanoparticles along the life cycle cannot be completely excluded a priori. In fact, freshwater could receive released (nano) materials leached from treated artefacts, and wastewater could receive end-of-life residues of the products, thus affecting both ecological and human targets. For these reasons, the water compartment is identified as one of the main concerns for the assessment of potential environmental impacts of such innovative products.

Accordingly, the first ITS tier (tier 1) is focused on the assessment of the acute aquatic toxicity, by applying a set of three bioassays aiming at the identification of the possible short-term effects generated by the release of the formulation into the aquatic environment (i.e. OECD standard method 202 with the crustacean *Daphnia magna* (OECD 2004), OECD 201 with the algae *Pseudokirchneriella subcapitata* (OECD 2011) and ISO 11348:2007 (ISO 2007) with the bacteria *Aliivibrio fischeri*). According to CLP regulation, only formulations with EC_50_ < 1 mg l^−1^ for at least one species are classified as acutely toxic (acute I). If this criterion is not met for any species, the toxicological testing should move to tier 2.

The second tier of the ITS focuses on the long-term (chronic) effects through the *D. magna* reproduction test, the standard OECD method 211 (OECD 1998). According to CLP regulation, formulations with NOEC in the range 0.1–1 mg l^−1^ are classified as “Chronic 3”, formulations with NOEC in the range 0.01–0.1 mg l^−1^ are classified as “Chronic 2”, and formulations with NOEC ≤ 0.01 are classified as “Chronic 1”, the more hazardous class concerning the long-term effects. If these criteria are not met, the toxicological testing should move to tier 3.

Finally, the third and last ITS tier is aimed at the exploration of possible effects that cannot be detected by applying acute and chronic toxicity test, such as cytotoxicity, DNA damage and mutagenicity. To this end, the set of bioassays has been expanded with the addition of the *umu*- and SOS Chromotest systems ISO 13829:2000 (ISO 2000), two short-term test systems based on the detection of chemically induced DNA lesions that could lead to DNA mutations or SOS response (bacterial error prone repair system) to bacterial strains that have been genetically engineered and providing screening information also on the genotoxic potential for human and ecological hazard assessment (OSPAR Commission 2002).

**Fig. 4:**
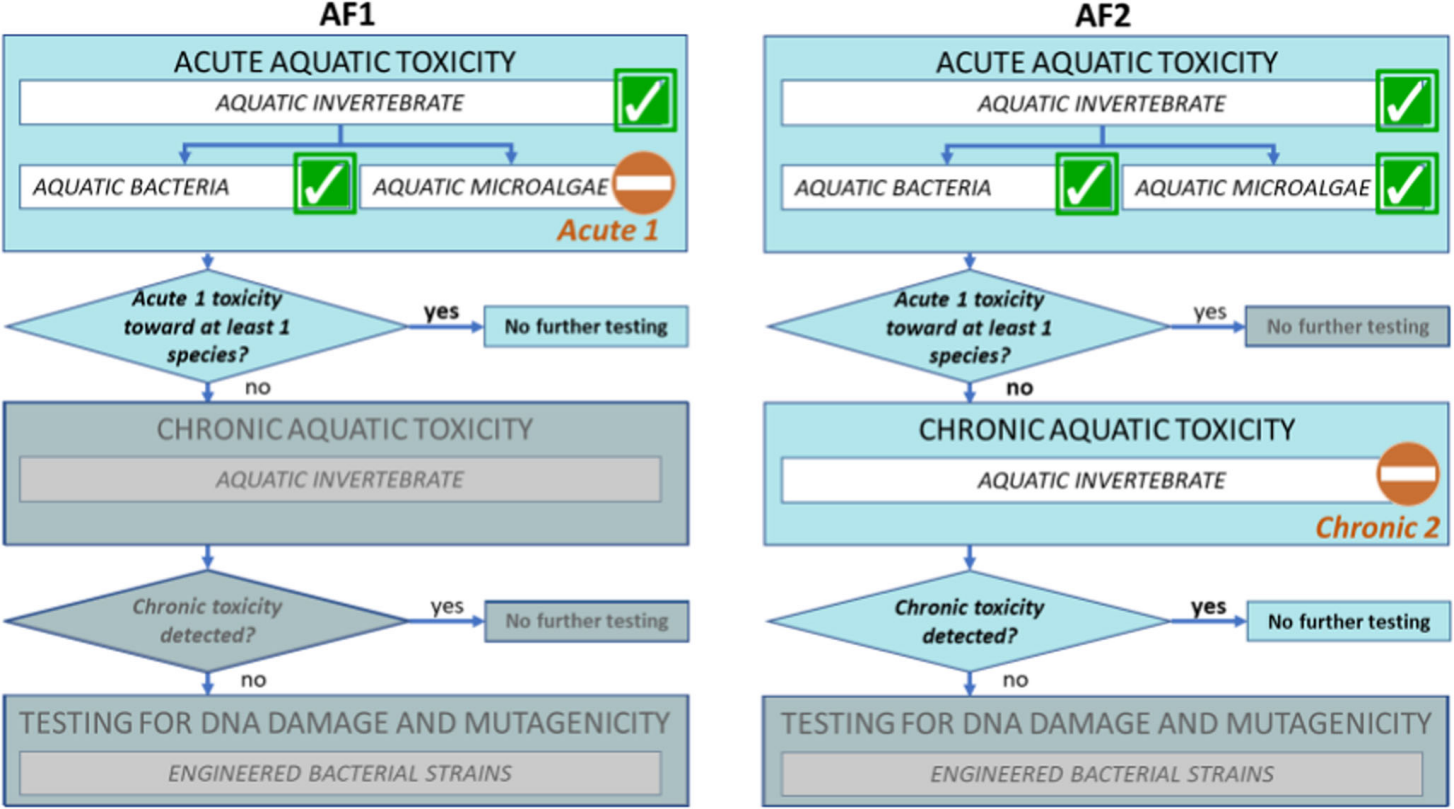
Results of step 3: advanced hazard assessment of adjusted formulations (AF1 and AF2)

Both AF1 and AF2 are therefore tested according to the described tiered ITS and the results show that (Fig. 4), while AF1 is classified as acute 1, due to an estimated EC50 < 1 mg l^−1^ for the algae *Pseudokirchneriella subcapitata*, AF2 is not acutely toxic (i.e. EC50 > 1 mg l^−1^ for all the three tested species); however, it shows a moderate chronic toxicity (i.e. 0.01 mg l^−1^ < NOEC ≤ 0.1 mg l^−1^) to the aquatic invertebrate *Daphnia magna*.

Accordingly, only AF2 is selected as the most promising innovative nano-based consolidant to be checked according to subsequent steps (safety and sustainability assessment). However, before moving to step 4 for safety assessment, exposure assessment must be carried out for both application ad post-application phases. As far as application phase is concerned, since usually nanomaterials represent only a very small percentage in the composition of innovative systems for conservation of works of art and therefore exposure in occupational settings (i.e. conservation studios and laboratories) is mainly driven by volatile compounds (e.g. solvents), it was decided to define four exposure classes according to the concentration (%*w*/*w*) of hazardous volatile components in the formulation. Specifically, exposure is classified as follows: negligible when the concentration of hazardous volatile components is lower than 1% (i.e. the cut-off value suggested also in CLP regulation) or when all recommended risk management measures (RMMs; e.g. gloves, goggles and suitable ventilation) are applied; low when the concentration of hazardous volatile components is between 1% and 10% and recommended RMMs are not applied; medium when the concentration is between 11% and 50% (with no RMMs); and finally, high when the concentration is higher than 50% (with no RMMs). Accordingly, since AF2 is composed by over 99% of water and the nano-ingredient 1, restorer’s exposure to it can be classified as negligible both with and without the application of suitable (and recommended) RMMs.

As far as the post-application phase is concerned, releases of formulations’ components (including nanomaterials) can be considered negligible or not significant because it is assumed that after application the consolidation system is bounded to the substrate and cannot be distinguished from it. For this reason, toxicity testing on released materials cannot be performed and safety assessment is carried out for the application phase only.

**Fig. 5:**
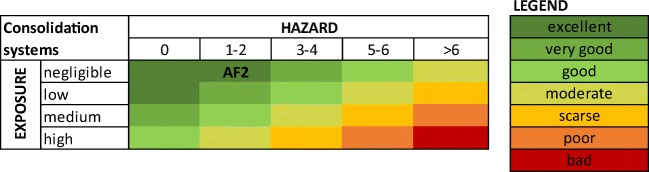
Results of step 4: safety assessment in the application phase of AF2

To this end (step 4), hazard and exposure data and information collected or generated in the previous steps are integrated according to the semi-quantitative control banding approach depicted in Fig. 5, where the results of hazard and exposure assessments are expressed according to five and four classes, respectively. The five hazard classes correspond to the number of human (H) and environmental (ENV) hazards assigned to a specific formulation according to the CLP self-classification approach. When formulation’s toxicity and ecotoxicity are also experimentally tested (as in step 3 of the sustainability assessment framework), these results can be used to adjust hazard classification in the matrix (e.g. by counting “hazard to the aquatic environment” if such environmental hazard is indicated by experimental tests performed on the formulation, although not assigned by CLP self-classification). The four exposure classes are those explained above (i.e. negligible, low, medium and high). By combining each of them in the control banding matrix, safety is assessed by using a simple colour code, from the greenest top left corner (i.e. excellent level of safety, which corresponds to a mixture with a concentration of hazardous volatile components up to 10% and classified for up to two health (H) and ENV hazards) to the reddest bottom right corner (i.e. bad level of safety, which corresponds to a mixture with a concentration of hazardous volatile components higher than 50% and classified for more than six H and ENV hazards).

Considering AF2, since exposure is negligible and the only assigned hazard is the chronic toxicity for aquatic environment, it is falling in the top safety class (i.e. excellent; see Fig. 5).

**Fig. 6:**
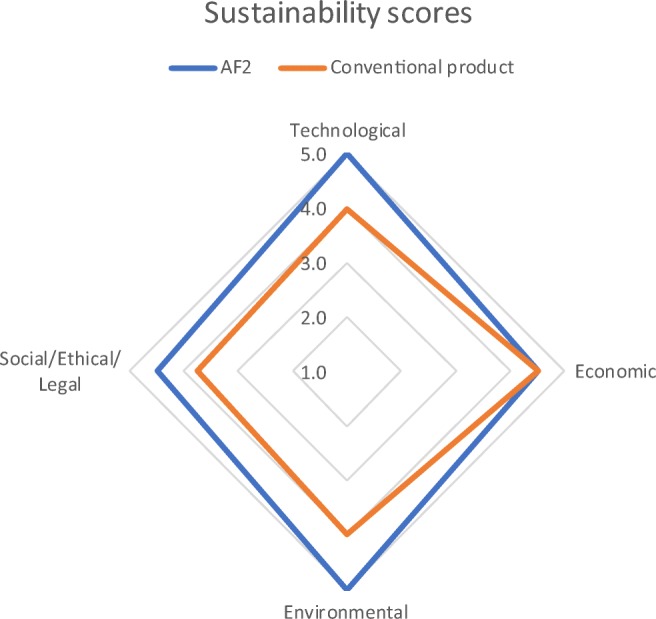
Results of step 5: sustainability assessment of AF2 and CP

Finally, the sustainability of AF2 is evaluated and compared with the one of conventional products (i.e. at least one benchmark consolidant already on the market, if existing), through a methodology based on MCDA, suitable to support the integration of heterogeneous data from different domains (step 5). Although such methodology is still under development, we can anticipate that it will integrate information from the three sustainability pillars (i.e. environment, economy and society), as well as information on technical features of the new products, described through semi-quantitative indicators (e.g. market size, regulatory barriers and compatibility). Under each pillar, indicators are scored according to five classes representing the level of satisfaction, from “excellent” to “bad”, of a specific product solution, i.e. 5 is assigned to “excellent”, 4 to “good”, 3 to “moderate”, 2 to “poor” and 1 to “bad”, and then mathematically aggregated to calculate an overall sustainability score. As an example of its application, Fig. 6 reports the sustainability scores obtained for AF2 and a conventional product (CP), showing that the overall sustainability of the innovative system is indeed very high and better than the CP’s one.

More specifically, according to the results of safety assessment, the environmental pillar reaches the highest score (i.e. 5) for AF2 while CP, composed by more hazardous ingredients and thus classified for a higher number of H and ENV hazards, lags behind; the same score is obtained by technological pillar, thus reflecting the highly satisfying performance of such an innovative consolidation system (AF2) compared with CP, the latter being characterised by worse efficiency and compatibility. Economic and social/ethical/legal pillars obtain slightly lower scores (i.e. between 4 and 5) for AF2 than the other two pillars, due to the need to further invest on its industrial upscale and on addressing easy to be solved regulatory barriers (i.e. formalising compliance to relevant legislation), respectively. However, by comparing them with those obtained by CP, while economic scores are equal (the reason is that although CP is already industrially upscaled, it has a higher market price), AF2 performs better than CP according to the social/ethical/legal pillar, thanks to the fact that it allows a complete re-treatability of the artworks and an excellent long-term action.

## Discussion

The proposed sustainability framework implementing the Safe by Design (SbD) concept allows to guide step by step product developers in the early design of sustainable innovative solutions for the conservation of works of art by suggesting a way to proceed towards sustainability along the Stage-Gate innovation process.

To this end, the triple bottom line (TBL) approach is adopted, although at a screening level, in the first two steps of the framework, and it is later expanded and fully covered by the fifth and last steps, where a MCDA-based methodology is applied to integrate environmental, economic, social/ethical/legal and technical aspects related to innovative products and to allow their comparison with conventional ones, if existing.

In between (steps 3 and 4), large space is devoted to investigating the environmental pillar of sustainability through the inclusion and implementation of the SbD concept. This is done by adopting a tiered approach, which includes both screening and advanced hazard assessments. While screening hazard assessment that is based on the use of information available in SDS and on the application of the CLP self-classification approach can always be applied, advanced hazard assessment requires a further investment on computational and/or experimental activities, which is more difficult to get at industrial level (particularly by small and medium enterprises because of resource constraints). However, it must be noted that, when dealing with mixtures containing nanoparticles, adequate physicochemical and (eco) toxicological information about these ingredients are not always available through SDS. Moreover, their behaviour can vary significantly according to the media in which they are embedded. For this reason, it is recommended to experimentally investigate how ingredients in the nanoform behave in the specific mixture and how this can drive exposure and (eco) toxicity to different targets. This would allow to reduce the uncertainty associated to the results of the screening assessment and therefore to obtain a more robust safety classification.

In the framework, human health and environmental safety is checked in the fourth step through semi-quantitative or quantitative approaches, according to data availability. Specifically, while for safety in the application phase, a control banding approach is proposed, which allows for the inclusion of appropriate RMMs, for safety in the post-application phase, a deterministic estimation is preferred, which allows to mathematically combine the previously derived predicted environmental concentration (PEC) and predicted no-effect concentration (PNEC).

Such flexibility of the framework allows its applicability in different contexts, by iterating and tailoring each step according to specific users’ (i.e. mainly product developers but also restorers) needs, thus facilitating the high interaction demanded in the field of conservation science. As far as available tools and information which can be used for applying the framework, the user can refer to the NanoReg2 SIA Toolbox (https://www.siatoolbox.com/) and to the two databases now accessible through the European Union Observatory for Nanomaterials (EUON): NanoData (https://nanodata.echa.europa.eu/) and eNanoMapper (https://euon.echa.europa.eu/enanomapper).

However, the proposed framework should not be considered restricted to cultural heritage science and to nano-based formulations only but can be extended to the development of innovative chemical products across various application domains, regardless of whether they occur as individual substances or in mixtures. The adjustment of the integrated testing strategy (ITS) used in safety assessment as well as of some specific criteria for sustainability assessment (e.g. criteria for the social/ethical/legal or technological pillars) will be the only requirement needed for using the sustainability assessment framework in other application contexts.

## Conclusions and future developments

The potential impacts on environment and human health of innovative nano-based products for conservation of works of art should be addressed already starting from the first stages of the innovation process, according to a SbD approach. The assessment of their safety should also be included in a more comprehensive assessment of their sustainability, suitable to evaluate and weight the environmental, economic and social implications of the new products as required under the authorisation and restriction provisions of the REACH regulation.

In this work, taking into account state-of-the-art approaches for safety and sustainability assessment of nanomaterials, a sustainability assessment framework implementing the SbD concept has been proposed with the aim of supporting product developers in the development of innovative and sustainable nano-based products for cultural heritage conservation. Its goal is to guide them in investing additional efforts in the design phase in order to limit the need to find better alternatives in the future, once the products are ready to enter or are already in the market. This could imply additional costs in the design phase (negligible for the screening hazard assessment while more relevant in case-specific ecotoxicological tests are performed within the advanced hazard assessment), which however would be lower than the personnel and economic resources to be spent for the identification of safer alternatives at later stages of the innovation process.

The example of application on a hypothetical (although realistic) case study allowed us to illustrate how the proposed framework can be applied in practice through structured methodological steps, which can be further tailored and iterated as long as it is needed in the decision-making process.

Application to real case studies is currently being finalised and will be presented in other papers (Semenzin et al. in preparation; Giubilato et al. in preparation). A set of nano-based products for cleaning, strengthening and protection has been developed, and their screening (i.e. CLP self-classification) and advanced (i.e. according to ITS) hazard assessments as well as human health and environmental safety assessment were completed. The development of the MCDA-based methodology for sustainability assessment is being completed, and its application will allow the evaluation of innovative products in terms of environmental, economic, social and technical performance as well as their comparison with conventional products if any.
